# Antigen Specific Humoral and Cellular Immunity Following SARS-CoV-2 Vaccination in ANCA-Associated Vasculitis Patients Receiving B-Cell Depleting Therapy

**DOI:** 10.3389/fimmu.2022.834981

**Published:** 2022-01-28

**Authors:** Paige K. Marty, Virginia P. Van Keulen, Courtney L. Erskine, Maleeha Shah, Amber Hummel, Michael Stachowitz, Samantha Fatis, Dane Granger, Matthew S. Block, Alí Duarte-García, Kenneth J. Warrington, Elitza S. Theel, Xian Zhou, Hu Zeng, Ulrich Specks, Patricio Escalante, Tobias Peikert

**Affiliations:** ^1^ Division of Pulmonary and Critical Care Medicine, Department of Medicine, Mayo Clinic, Rochester, MN, United States; ^2^ Department of Immunology, Mayo Clinic, Rochester, MN, United States; ^3^ Division of Clinical Microbiology, Department of Laboratory Medicine and Pathology, Mayo Clinic, Rochester, MN, United States; ^4^ Department of Oncology, Mayo Clinic, Rochester, MN, United States; ^5^ Division of Rheumatology, Department of Medicine, Mayo Clinic, Rochester, MN, United States

**Keywords:** COVID-19 vaccination, rituximab, ANCA-associated vasculitis, cellular immune response, humoral immune response

## Abstract

Humoral vaccine responses are known to be suboptimal in patients receiving B-cell targeted therapy, and little is known about vaccine induced T-cell immunity in these patients. In this study, we characterized humoral and cellular antigen-specific anti-SARS-CoV2 responses following COVID-19 vaccination in patients with ANCA-associated vasculitis (AAV) receiving anti-CD20 therapy, who were either B-cell depleted, or B-cell recovered at the time of vaccination and in normal control subjects. SARS-CoV-2 anti-spike (S) and anti-nucleocapsid (NC) antibodies were measured using electrochemiluminescence immunoassays, while SARS-CoV-2 specific T-cell responses to S glycoprotein subunits 1 (S1) and 2 (S2) and receptor binding domain peptide pools were measured using interferon-gamma enzyme-linked immunosorbent spot (ELISPOT) assays. In total, 26 recently vaccinated subjects were studied. Despite the lack of a measurable humoral immune response, B-cell depleted patients mounted a similar vaccine induced antigen-specific T-cell response compared to B-cell recovered patients and normal controls. Our data indicate that to assure a humoral response in patients receiving anti-CD20 therapy, SARS-CoV-2 vaccination should ideally be delayed until B-cell recovery (CD-20 positive B-cells > 10/μl). Nevertheless, SARS-CoV-2 vaccination elicits robust, potentially protective cellular immune responses in these subjects. Further research to characterize the durability and protective effect of vaccine-induced anti-SARS-CoV-2 specific T-cell immunity are needed.

## Introduction

The unknown efficacy of vaccination against severe acute respiratory syndrome coronavirus 2 (SARS-CoV-2), the cause of Coronavirus Disease 2019 (COVID-19), in patients treated with anti-CD20 agents (e.g., rituximab and obinutuzumab) represents an ongoing challenge. The American College of Rheumatology (ACR) currently recommends that patients on rituximab (RTX) therapy receive COVID-19 vaccination four weeks prior to the next scheduled treatment cycle and for the next therapy to be delayed until 2 to 4 weeks after the final vaccine dose (1). Similarly, the European Alliance of Associations for Rheumatology (EULAR) also recommends individualizing COVID-19 vaccination to “optimize immunogenicity” in patients on RTX; for example, vaccinating 6 months following the last administration of the medication and 4 weeks prior to the next dose (2).

Several studies have investigated the outcomes of COVID-19 in patients with rheumatologic diseases. Glucocorticoid use equivalent to ≥10 mg of prednisone/day was associated with increased odds of hospitalization related to COVID-19, whereas these odds were lower in patients receiving anti-TNF therapy (3). Several factors, including male sex, chronic heart or lung disease, moderate-to-high disease activity, and use of RTX, have been associated with COVID-19-related death in these patients (4). In a large registry study, the risk of severe COVID-19 (i.e., admission to the intensive care unit or death) and length of hospital stay have been found to be increased in patients on RTX after adjustment for confounding factors such as age, sex, and comorbidities (5). In addition, the shorter time from last RTX infusion to COVID-19 in patients with severe COVID-19 compared to those with mild or moderate disease suggests that these observed effects are linked to RTX induced B-cell depletion (5). These detrimental effects have been attributed to a lack of newly induced antigen-specific humoral immunity in these patients (6). This absence of antigen-specific antibodies has also been associated with chronic persistent SARS-CoV-2 infections requiring treatment with high-titer convalescent plasma for the clearance of the infection in a small subset of patients (7, 8). However, in contrast, the majority of B-cell depleted patients recover normally after COVID-19, suggesting that cellular immunity is sufficient to effectively clear the virus (9).

Immunosuppressive medications have been shown to alter host responses to several vaccines. In individuals with rheumatoid arthritis, methotrexate led to decreased humoral immune response (antibody titers 3 to 6 weeks following vaccination) to the pneumococcal vaccine (10). In the same analysis, patients on RTX also had reduced humoral immune responses to both pneumococcal and influenza vaccines suggesting decreased vaccine efficacy.

For the SARS-CoV-2 vaccine, anti-spike IgG titers are typically measured to assess vaccine efficacy. These titers were found to be lower in patients receiving B-cell depletion therapy, including RTX, compared to control individuals not receiving immunosuppressive therapy (11). This effect has been seen more prominently in patients who had received B-cell depletion therapy within the previous 6 months, indicating a link to the degree of B-cell depletion (11, 12). Patients on RTX have also been shown to have reduced humoral immune response following a single dose of SARS-CoV-2 mRNA vaccine (13), with further data suggesting that patients with inflammatory diseases on RTX have a negative antibody response following two doses of mRNA vaccine (14, 15). This phenomenon has also been observed in patients on anti-CD20 therapy for ANCA-associated vasculitis (AAV), multiple sclerosis or neuromyelitis optica spectrum disorders (16, 17).

Since previous data has suggested a blunted humoral immune response, T-cell immune responses to SARS-CoV-2 vaccines are of particular interest in patients receiving B-cell depleting therapy. Cellular immunity has been analyzed in various other vaccines. Patients with rheumatoid arthritis treated with RTX have been found to have lower humoral immunity to the influenza vaccine compared to those on other disease-modifying anti-rheumatic drugs (DMARDs). However, no differences in cellular immunity as demonstrated by influenza-specific CD4+ T-cells were observed (18). With regards to the SARS-CoV-2 vaccines, prospective studies comprehensively evaluating vaccine induced antigen-specific T-cell responses in patients primarily treated with RTX are lacking. Prior studies investigating patients receiving RTX for a variety of inflammatory rheumatologic conditions have reported humoral immune responses positively correlating to the level of B-cell depletion (16, 19). In addition, these studies also reported measurable antigen-specific T-cell responses in subsets of patients ranging between 28-58%. There was no detectable correlation between antigen-specific humoral and cellular immunity (8, 16, 19).

The objective of the present study was to quantify SARS-CoV-2 vaccine induced antigen-specific T-cell immunity and anti-SARS-CoV-2 humoral immune responses in patients with AAV treated with RTX who received the vaccine while B-cell depleted (CD20 positive B-cells < 10 cells/µl at the time of vaccination) or after recovery of B-cell counts (CD20 positive B-cells ≥ 10 cells/µl at the time of vaccination) and in normal controls without prior SARS-CoV-2 infection.

## Methods

### Study Population

Between April and August 2021, we prospectively enrolled patients with AAV at Mayo Clinic in Rochester, MN who were previously treated with rituximab and had received a complete SARS-CoV-2 vaccine series. Serum and peripheral blood mononuclear cells (PBMCs) were obtained from these patients and otherwise healthy control subjects fully vaccinated against SARS-CoV-2. Patients with AAV were classified as either B-cell depleted (< 10 CD20 positive B-cells/µl) or B-cell recovered (≥ 10 CD20 positive B-cells/µl) at the time of SARS-CoV-2 vaccination. The collection of PBMCs, serum samples, and clinical information was approved by our Institutional Review Board under protocol #13-004041 with written informed consent obtained from all subjects.

### SARS-CoV-2 Serologic Assays

Total antibodies against the nucleocapsid (NC) and receptor-binding domain (RBD) on subunit 1 (S1) of the spike (S) glycoprotein were detected in sera using the Roche Elecsys anti-SARS-CoV-2 and anti-SARS-CoV-2 S electrochemiluminescence immunoassays (ECLIAs; Roche Diagnostics, Indianapolis, IN), respectively. Both assays have received Emergency Use Authorization from the Food and Drug Administration and were performed and interpreted per manufacturer instructions for use (20). The anti-NC ECLIA is a qualitative assay with index values ≥1.1 considered positive. The anti-RBD ECLIA is semi-quantitative with values ≥0.8 U/mL considered positive and the analytical measurement range between 0.4 U/mL and 2500 U/mL.

### IFN-γ ELISPOT Assay

PBMCs were stimulated with recombinant SARS-CoV-2 S1 and S2 proteins, and peptide pools from SARS-CoV-2 S1 and S2 subunits and receptor binding domain (RBD) (Jet Peptide Technologies: PM-WCPV-S, PepMix™ SARS-CoV-2 spike glycoprotein, crude; and PM-WCPV-S-RBD, PepMix™ SARS-CoV-2 S-RBD), and tetanus toxoid (positive control; List Biological product #191A) and media (negative controls). Cryopreserved PBMC samples (2.5 x 10^5^ cells per condition) were thawed and analyzed in triplicate for the presence of interferon-γ (IFN-γ) positive spots (sfu). The plates were read on an AID EliSpot reader (Autoimmun Diagnostika GmbH, Strassberg, Germany). The number of IFN-γ positive spots were compared among vaccinated patients with AAV who were B-cell depleted or B-cell recovered and vaccinated healthy controls. Subjects were considered to have a positive response when the number of IFN-γ ELISPOT spots was greater than the median plus 2 times the standard deviation of the 3 media control wells for each individual (21).

### Flow Cytometry

Cryopreserved PBMCs were thawed and stained with antibodies or viability dyes listed in **Supplementary Table 1**. Samples were acquired on a 4-laser Attune NxT flow cytometer (ThermoFisher Scientific). Up to 1.1 × 10^6^ PBMCs were acquired for each sample. All samples were acquired on the same day with consistent settings. Data were analyzed with FlowJo 10.8.1 (Becton Dickinson).

### Statistical Analysis

The statistical analysis was performed using the PRISM 5 software (GraphPad). Continuous variables were compared using the unpaired Mann-Whitney test, T-test or 1-way ANOVA. Categorical variables were compared using the Chi-square test. P-values < 0.05 were considered statistically significant.

## Results

A total of 26 recently vaccinated individuals, including 11 B-cell depleted and 8 B-cell recovered patients with AAV (all previously treated with RTX) and 7 healthy volunteers, were studied. Demographic and vaccine related information is summarized in **Table 1**. Seventeen of the patients with AAV had a diagnosis of Granulomatosis with Polyangiitis (GPA). One patient had Eosinophilic Granulomatosis with Polyangiitis (EGPA), and one had GPA/EGPA overlap. While all B-cell recovered patients were off immunosuppression and on remission maintenance therapy, 8 of the B-cell depleted patients were on low-dose (n=6) and 2 were on high-dose prednisone (remission induction therapy) in addition to the RTX. The majority of participants (17/26, 65%) received the BioNTech/Pfizer BNT162b2 vaccine, while the remainder received either Johnson & Johnson/Janssen Ad26.COV.2.S (n=1) or Moderna mRNA-1273 (n=9). The time from the final dose of vaccine to study blood sample collection was similar between B-cell depleted and recovered patients but was slightly longer for healthy volunteers. (**Table 1**)

**Table 1:** Subject Characteristics.

	B-cell Depleted Patients (< 10 CD20 positive B-cells/μl)	B-cell Recovered Patients (≥ 10 CD20 positive B-cells/μl)	Normal Subjects	p-value
**Age** years, mean (range)	51 (24-73)	58 (39-67)	55 (42-74)	NS
**Gender**				
Women	5	4	7	NS
Men	6	4	1	
**COVID-19 Vaccine**				
Pfizer	4	7	5	NS
Moderna	6	0	2	
J&J	1	1	0	
**Time from Vaccination to Sampling** days, mean (Range)	47 (9-146)	63 (8-126)	136 (83-194)	0.005 B-cell depleted versus B-cell recovered p=NS
**Other Immunosuppression** Prednisone n (median mg/day (range))	8 (7.25 (5-60))	0	N/A	0.007
Plus Mepolizumab	2	0		
None	3	8		
**Therapeutic Phase**				
Remission Induction	2	0	N/A	NS
Remission Maintenance	9	8		
**Diagnosis**				
GPA	9	8	N/A	NS
EGPA	1	0		
GPA/EGPA	1	0		

N/A, not applicable; NS, not significant.

None of the study participants had prior documented COVID-19 infection, and at the time of study enrollment, all subjects were negative for SARS-CoV-2 anti-NC antibodies (16, 22, 23).

**Figure 1:**
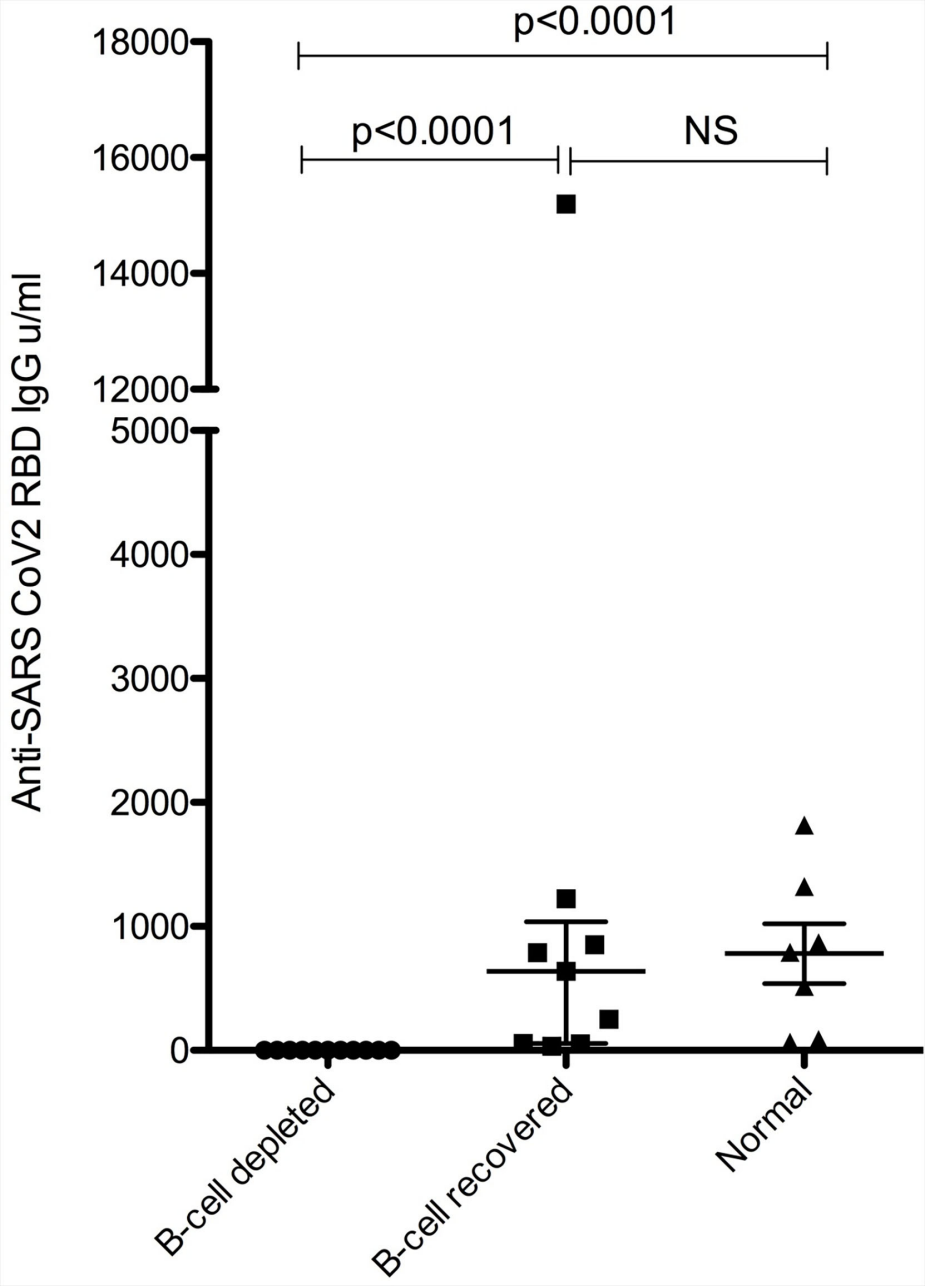
Anti-SARS-CoV-2 RBD IgG levels. NS, not significant.

None of the vaccinated B-cell depleted patients were positive for SARS-CoV-2 RBD (i.e. Spike) total antibodies (<0.4 U/mL); however, all B-cell recovered patients and normal subjects had a positive anti-RBD antibody response, and antibody levels were similar between B-cell recovered patients with AAV and healthy vaccinated individuals. (**Figure 1**).

**Figure 2:**
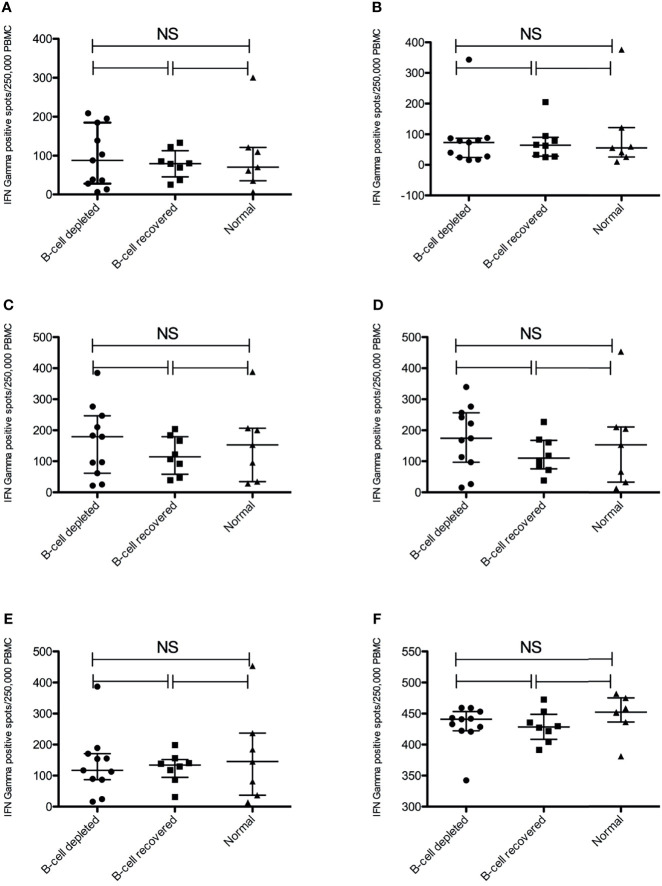
Anti-SARS-CoV-2 Spike T-cell responses measured by IFNγ ELISPOT positive spots using different antigens. **(A)** S1 subunit SARS-COV-2 spike glycoprotein **(B)** S2 subunit SARS-COV-2 SPIKE, **(C)** SARS-COV-2 SPIKE peptide pool (JPT v1-158P), **(D)** SARS-COV-2 spike peptide pool (JPT v2-157P), **(E)** SARS-COV-2 spike RBD peptide pool and **(F)** Tetanus Toxoid. NS, not significant.

**Figure 3:**
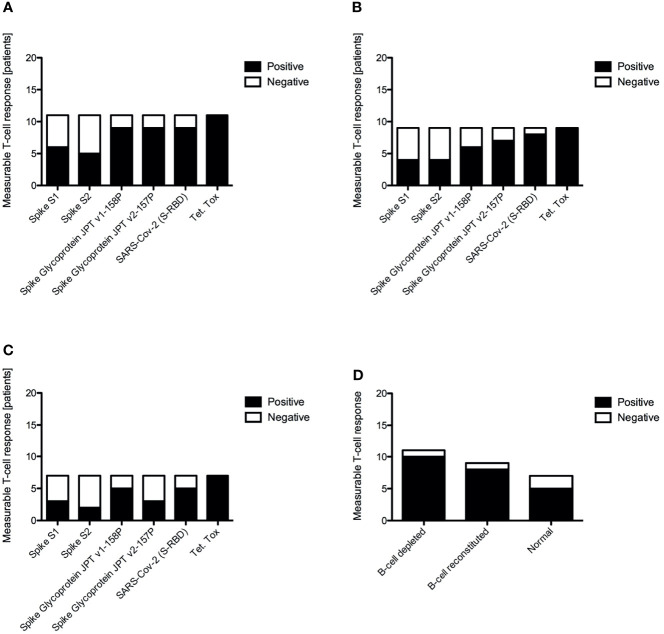
Positive versus negative vaccine induced antigen specific T-cell responses to different antigens by IFNγ ELISPOT for B-cell depleted AAV patients **(A)**, B-cell recovered AAV patients **(B)**, healthy control subjects **(C)** and overall summary of a positive measurable T-cell response across all tested antigens **(D)**.

Antigen-specific T-cell responses targeting the SARS-CoV2 S protein were measured by ELISPOT using recombinant S1 and S2 subunits and three established S glycoprotein peptide pools. Tetanus toxoid and culture media served as the positive and negative controls, respectively. All subjects had a strong IFN-γ response to tetanus toxoid. Despite the lack of a measurable humoral immune response, B-cell depleted patients with AAV were able to produce similar antigen-specific IFN-γ positive response targeting the S glycoprotein peptide pools compared to B-cell recovered patients and healthy controls. In fact, there were no statistically significant differences across the groups (**Figure 2**). Most individuals produced a positive IFN-γ response to at least one of the S-specific antigens, including 10 of the B-cell depleted (90.9%) and 8 of the B-cell recovered patients with AAV patients (88.9%), as well as 5 of the healthy control subjects (71.4%). (**Figure 3** and **Supplementary Figures 1**–**3**) There were no significant differences in antibody and T-cell response between those who received the BioNTech/Pfizer BNT162b2 and those who received Moderna mRNA-1273 among B-cell depleted individuals and controls (**Supplementary Figures 4**, **5**). Since none of the B-cell recovered patients received the Moderna mRNA-1273 vaccine, vaccines could not be compared for this group.

**Figure 4:**
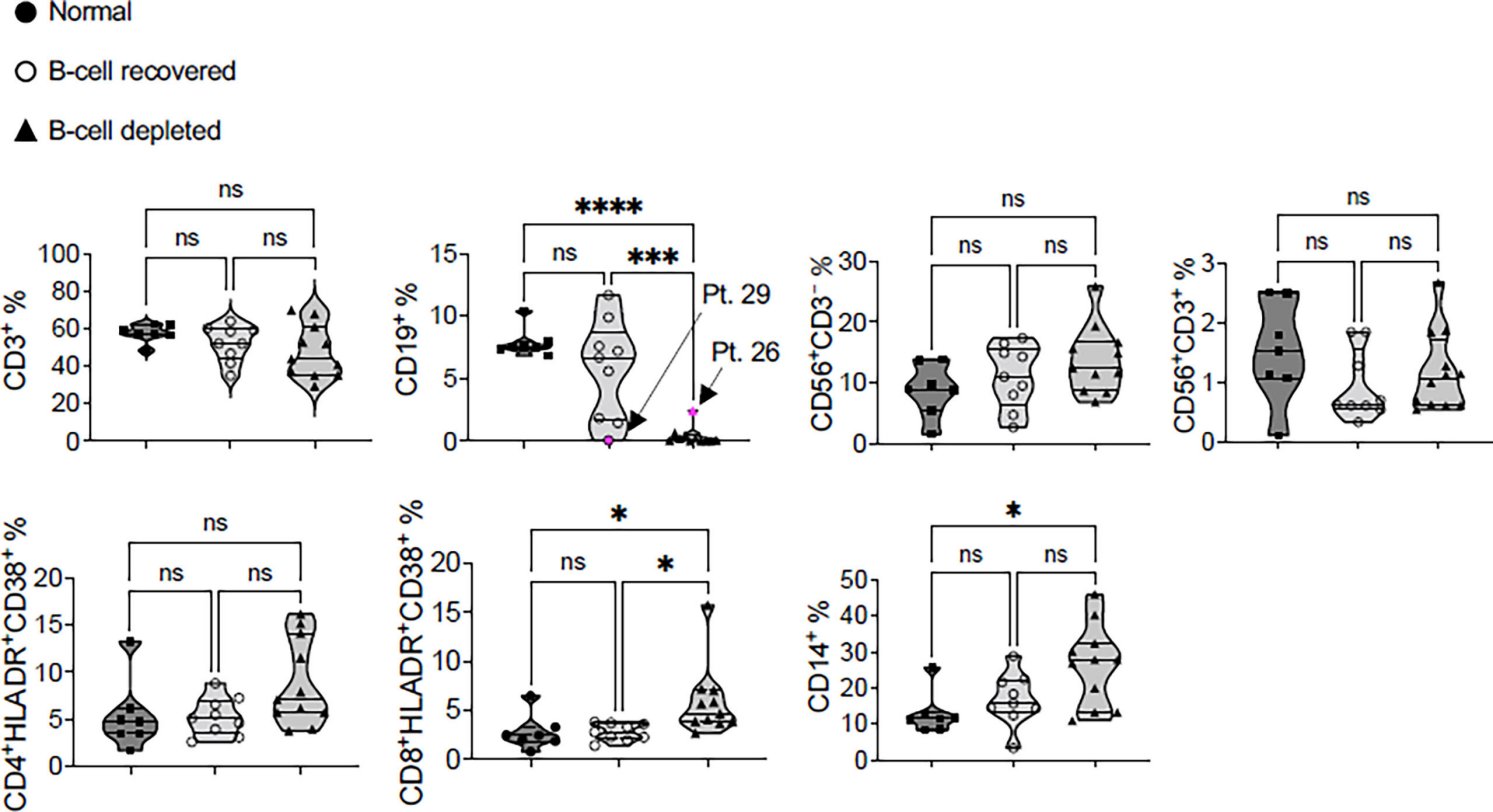
Immunophenotyping of the post-SARS-CoV-2 vaccine PBMC samples of B-cell depleted, B-cell recovered AAV patients and healthy controls. Patient #29 is a B-cell recovered patient who was retreated with rituximab after completing the COVID vaccine and patient #26 recovered B-cells following the vaccination. All statistics were done using one-way ANOVA. *P < 0.05, ***P < 0.01, ****P < 0.001. NS, not significant.

To further characterize the IFN-γ ELISPOT results, we analyzed potential differences in immune cell subsets within the PBMC collected from B-cell depleted and B-cell recovered patients with AAV and normal controls using FACS. This analysis was done at the time of blood sampling, after vaccination. In the majority of patients, FACS confirmed the absence or presence of B-cells (CD19 positive cells) according to the original group assignments at the time of vaccination. However, in two patients, #26 (B-cell depleted) and #29 (B-cell recovered), the B-cell status changed based on subsequent B-cell recovery or need for repeat rituximab treatment (**Figure 4**). There were no significant differences among the groups relative to the presence of CD3+ T-cells, CD56+CD3– natural killer (NK) cells, CD56+CD3+ NKT cells, and TCR-activated CD4+ T-cells (CD4+HLADR+CD38+) between B-cell depleted, B-cell recovered patients with AAV and normal controls. In contrast, B-cell depleted patients with AAV had more TCR-activated CD8+ T-cells (CD8+HLADR+CD38+) and CD14+ monocytes compared to B-cell recovered patient with AAV and normal controls, respectively (**Figure 4**). This suggests a slight skewing of the circulating immune cell populations among our study groups, likely a consequence of B-cell depletion.

## Discussion

Our study clearly demonstrates that B-cell depleted patients with < 10/µl circulating CD20 positive B-cells are unable to generate an elevated antibody response following SARS-CoV-2 vaccination, irrespective of vaccine manufacturer. Delaying SARS-CoV-2 vaccination until after B-cell recovery (i.e., >10/µl circulating CD20 positive B-cells) completely restored the ability of previously B-cell depleted patients to generate a humoral immune response against SARS-CoV-2 compared to normal control subjects. These observations are consistent with prior observations and highlight the importance of B-lymphocytes for the development of vaccine-induced humoral immunity in infection-naïve individuals (24, 25). Our findings support current guidelines that clinical monitoring of circulating B-cells may be very valuable to individualize the optimal timing of SARS-CoV-2 vaccination in patients receiving anti-CD20 therapy (26, 27). While the patients enrolled into this study had a diagnosis of AAV, a similar impact of B-cell depletion on vaccine induced humoral immunity has also been documented across various other diseases managed with B-cell depletion, including B-cell non-Hodgkin lymphoma, multiple sclerosis, and rheumatoid arthritis (17, 28, 29).

While previous studies have largely focused on the impact of rituximab on antibody production, there is increasing evidence suggesting the SARS-CoV-2 specific T-cell immunity is also important for adequate response to infection and vaccination (12, 30). Prendecki and colleagues recently studied humoral and cellular immune responses to both the Pfizer and AstraZeneca SARS-CoV-2 vaccines, among 119 immunosuppressed patients in the United Kingdom (12). Among these heterogenous patients 59.3% and 82.6% of patients developed vaccine induced humoral and cellular immune responses, respectively. T-cell responses were measured in 46/91 SARS-CoV-2 naïve patients and a detectable response was identified in 38/46 (82.6%) of these patients using an IFN-γ ELISPOT assay. T-cell responses were similar between patients irrespective of the humoral immune response. The investigators measured vaccine immunity in 30 COVID-19 naïve, B-cell depleted patients. While 18 of these patients had no detectable antibody response, 15/18 (83.3%) had a detectable T-cell response, indicating a much larger impact of B-cell depletion on humoral rather than cellular immunity. In another recent publication, Mrak and colleagues reported their analysis of B- and T-cell responses to COVID-19 vaccination among 74 rituximab treated patients who were vaccinated with two doses of a mRNA-vaccine (30). Seroconversion was seen in 39% of patients, however this included only 1/36 B-cell depleted patients. Interestingly a T-cell response to the S1 subunit of the SARS-CoV-2 S glycoprotein was only detectable in 26/45 patients (58%) by IFN-γ ELISPOT, and the magnitude of the response was lower compared to healthy controls. T-cell responses were again similar between patients who did or did not seroconvert. Interestingly, in contrast to our study, a large proportion of the patients included in these two studies were also on other disease modifying medications, in addition to the rituximab (12, 16). Among our patients, only 8/19 (42%) patients with AAV were on other immunosuppressive medications, primarily low dose prednisone with only 2 patients taking > 10 mg/day.

In our study nearly all B-cell depleted patients with AAV (91%) generated a measurable SARS-CoV-2 specific vaccine-induced T cell response. The frequency and magnitude of these responses were similar to those observed in B-cell recovered, rituximab treated patients with AAV and among healthy controls. This confirms the independence of vaccine-induced T-cell responses from the presence of B-lymphocytes. These vaccine-induced T-cell responses may provide a level of protection from COVID-19 despite the absence of a humoral immune response. We identified only one B-cell depleted patient (#23) who was not considered to have a positive T-cell response (21). This individual received the BioNTech/Pfizer BNT162b2 vaccine at the beginning of remission-induction therapy with rituximab and high-dose corticosteroids (60 mg of prednisone daily). The lack of T-cell response in this patient was unlikely to be explained solely by the high dose glucocorticoid therapy as another B-cell depleted patient (#9) treated with rituximab and 60 mg of prednisone daily developed a notable T-cell response. Interestingly, patient #23 developed COVID-19 five months post-vaccination, suggesting clinically ineffective immunization.

Our results are of significant clinical importance and should be used to inform discussions between clinicians and B-cell depleted patients about the optimal timing of SARS-CoV-2 vaccination to maximize effectiveness. Our data affirms that while patients without B-cells will not develop anti-Spike RBD antibodies (11), this response will return if vaccination occurs following B-cells recovered. Consequently, patients should be vaccinated after B-cell recovery. In patients who are unable to interrupt B-cell depleting therapy to allow recovery due to continued activity of the underlying disease, vaccination may still elicit a normal antigen specific T-cell response against the SARS-CoV-2 S glycoprotein, even in the presence of low dose glucocorticoid therapy. While this response almost certainly confers a better level of protection compared to no vaccination, the exact level of protection against COVID-19 remains unclear.

The main limitation of our study is the relatively small sample size, which was due to our goal to recruit a carefully selected study population to best evaluate the impact of B-cell depletion on cellular immunity in response to SARS-CoV-2 vaccination. In contrast to previous studies, this approach enabled us to apply established immune profiling techniques to all enrolled subjects (23, 31, 32). Additionally, these samples were obtained from a biorepository study as opposed to an ongoing prospective trial. Therefore, longitudinal T-cell responses could not be assessed. Another potential limitation was the slightly longer delay in post-vaccination sampling between the normal control subjects and the B-cell depleted and recovered patients, respectively. While this could provide a potential explanation for the slightly lower proportion of normal subjects with a positive T-cell response (71.4% compared to 88.9% in the B-cell recovered group and 90.9% in the B-cell depleted group), this potential limitation does not impact the conclusions from our study. The exact duration of SARS-CoV-2 mRNA vaccine-induced T-cell responses remain unknown, although T-cell responses have been shown to persist for at least 10 months for SARS-CoV-2 and for up to two years in patients with SARS-CoV-1 (33). Since we used whole PBMCs we cannot tell which specific cell types are producing the IFN-γ, and thus this may be originating from other immune cells, such as NK cells. However, both ELISPOT and intracellular cytokine staining have been utilized to measure antigen-specific T-cell response to vaccination (21, 34, 35). Further studies should include more detailed characterization of the IFN-γ secreting cells in this patient population.

While B-cell depleted patients are unable to produce antibody responses following SARS-CoV-2 vaccination, these humoral responses are restored following B-cell recovery. Despite a lack of humoral vaccine immunity, most of these patients demonstrated robust vaccine antigen-specific T-cell responses to SARS-CoV-2 S epitopes. While further studies determining the effectiveness and optimal timing of COVID-19 vaccination in patients on B-cell depleting therapies are needed, our data suggests that these individuals should be vaccinated following B-cell recovery to elicit optimal humoral and cellular responses against SARS-CoV-2.

## Data Availability Statement

The original contributions presented in the study are included in the article/**Supplementary Material**. Further inquiries can be directed to the corresponding author.

## Ethics Statement

The studies involving human participants were reviewed and approved by Institutional Review Board at Mayo Clinic. The patients/participants provided their written informed consent to participate in this study.

## Author Contributions

Conception and design, acquisition of data, analysis, and interpretation of data: PM, VV, CE, MaS, AH, MiS, SF, ET, DG, XZ, HZ, US, PE, TP, MB, AD-G, and KW. Drafting and revision of the article: PM, VV, CE, MaS, MiS, ET, HZ, US, PE, and TP. Final approval of the version to be submitted: PM, VV, CE, MaS, ET, AH, MiS, SF, DG, HZ, US, PE, TP, XZ, MB, AD-G, and KW.

## Funding

Funding was provided internally through the Mayo Clinic.

## Author Disclaimer

This paper’s contents are solely the responsibility of the authors and do not necessarily represent the official views of the National Institutes of Health, Mayo Clinic, or any organization.

## Conflict of Interest

PE research work has been supported by the National Institute of Allergy and Infectious Diseases at the National Institutes of Health [AI141591] and Mayo internal research grant. PE, TP, and their institution have filed two patent applications related to immunodiagnostic laboratory methodologies for latent tuberculosis infection (Patent numbers: 9678071 and 10401360). To date, there has been no income or royalties associated with those filed patent applications. PE participated in a short-term advisory scientific board for DiaSorin Molecular in 2020, which was outside the scope of the submitted manuscript, and honorarium was paid to his institution. US has received research grant support from Genentech. HZ work has been supported by the National Institute of Arthritis, Musculoskeletal, and Skin Diseases at the National Institutes of Health [Award Number R01AR077518]. ET sat on an advisory board for Roche Diagnostics within the last 24 months and is an advisory board member for EuroImmun US, Serimmune Inc., and Oxford Immunotech. MB has received institutional research support from Bristol-Myers Squibb, Genentech, Immune Design, Pharmacyclics, Marker Therapeutics, Sorrento Therapeutics, Transgene, Viewpoint Molecular Targeting, Merck and TILT Biotherapeutics. MB is also an unpaid Scientific Advisor for Sorrento Therapeutics, Viewpoint Molecular Targeting, and TILT Biotherapeutics. AG has received research grants from the Rheumatology Research Foundation and the Centers for Disease Control and Prevention. KW has received clinical trial support from Eli Lilly and Kiniksa and honoraria from Chemocentryx.

The remaining authors declare that the research was conducted in the absence of any commercial or financial relationships that could be construed as a potential conflict of interest.

## Publisher’s Note

All claims expressed in this article are solely those of the authors and do not necessarily represent those of their affiliated organizations, or those of the publisher, the editors and the reviewers. Any product that may be evaluated in this article, or claim that may be made by its manufacturer, is not guaranteed or endorsed by the publisher.

## Supplementary Material

Supplementary Figure 4 Anti-SARS-CoV-2 RBD IgG levels by vaccine type.

Supplementary Figure 5 Anti-SARS-CoV-2 Spike T-cell responses measured by IFNγ ELISPOT positive spots using different antigens in B-cell depleted patients and normal subjects broken down by vaccine type. **(A).** S1 subunit SARS-COV-2 spike glycoprotein **(B)**. S2 subunit SARS-COV-2 SPIKE, **(C).** SARS-COV-2 SPIKE peptide pool (JPT v1-158P), **(D).** SARS-COV-2 spike peptide pool (JPT v2-157P), **(E).** SARS-COV-2 spike RBD peptide pool and **(F)** Tetanus Toxoid.
