# Supplementary material for: Antigen Specific Humoral and Cellular Immunity Following SARS-CoV-2 Vaccination in ANCA-Associated Vasculitis Patients Receiving B-Cell Depleting Therapy
Source: Front Immunol. 2022 Jan 28;13:834981. doi: 10.3389/fimmu.2022.834981 (PMC8831839; doi:10.3389/fimmu.2022.834981)
Supplement: Supplementary file 1 [file Table_1.docx]

Supplemental Table 1: Antibodies and Fluorochromes used in Flow Cytometry Analysis

| **Antibody target** | **Fluorochrome conjugate** | **Clone** | **Supplier** | **Catalog #** |
| --- | --- | --- | --- | --- |
| CD56 | FITC | NCAM16.2 | BD Biosciences | 340723 |
| CD20 | PE | 2H7 | Biolegend | 302306 |
| HLA-DR | PerCP | AC122 | Miltenyi | 130-095-291 |
| Fixable Viability Stain 620 |  |  | BD Biosciences | 564996 |
| CD185 | Alexa Fluor 647 | J252D4 | Biolegend | 356905 |
| CD4 | Alexa Fluor 700 | RPA-T4 | Biolegend | 300526 |
| CD45RA | APC-Cy7 | HI100 | Tonbo Biosciences | 25-0458-T100 |
| CD197 | PE-Cy7 | G043H7 | Biolegend | 353226 |
| CD38 | Brilliant Violet 421 | HIT2 | Biolegend | 303525 |
| CD8a | Brilliant Violet 510 | RPA-T8 | Biolegend | 301048 |
| CD127 | Brilliant Violet 605 | A019D5 | Biolegend | 351334 |
| CD3 | Super Bright 645 | OKT3 | Life Technologies | 64-0037-42 |
| CD279 | Brilliant Violet 711 | EH12.2H7 | Biolegend | 329928 |
| CD25 | Brilliant Violet 785 | BC96 | Biolegend | 302638 |
| IgD | FITC | IA6-2 | Biolegend | 348206 |
| CD24 | PE | ML5 | Biolegend | 311106 |
| IgM | PerCP/Cyanine5.5 | MHM-88 | Biolegend | 314512 |
| CD21 | APC | B-ly4 | BD Biosciences | 561357 |
| CD11c | RedFluor 710 | 3.9 | Tonbo | 80-0116-T025 |
| CD138 | APC-Cy7 | MI15 | Biolegend | 356528 |
| CD27 | PE-Cy7 | M-T271 | Biolegend | 356412 |
| CD38 | Brilliant Violet 421 | HIT2 | Biolegend | 303525 |
| Ghost Dye Violet 510 |  |  | Tonbo Biosciences | 13-0870-T500 |
| CD3 | Super Bright 600 | OKT3 | Thermo Fisher | 63-0037-42 |
| CD19 | Briliant Violet 650 | HIB19 | Biolegend | 302238 |
| CD14 | Brilliant Violet 785 | M5E2 | Biolegend | 301840 |
