# Supplementary figures and images for: Antigen Specific Humoral and Cellular Immunity Following SARS-CoV-2 Vaccination in ANCA-Associated Vasculitis Patients Receiving B-Cell Depleting Therapy
Source: Front Immunol. 2022 Jan 28;13:834981. doi: 10.3389/fimmu.2022.834981 (PMC8831839; doi:10.3389/fimmu.2022.834981)

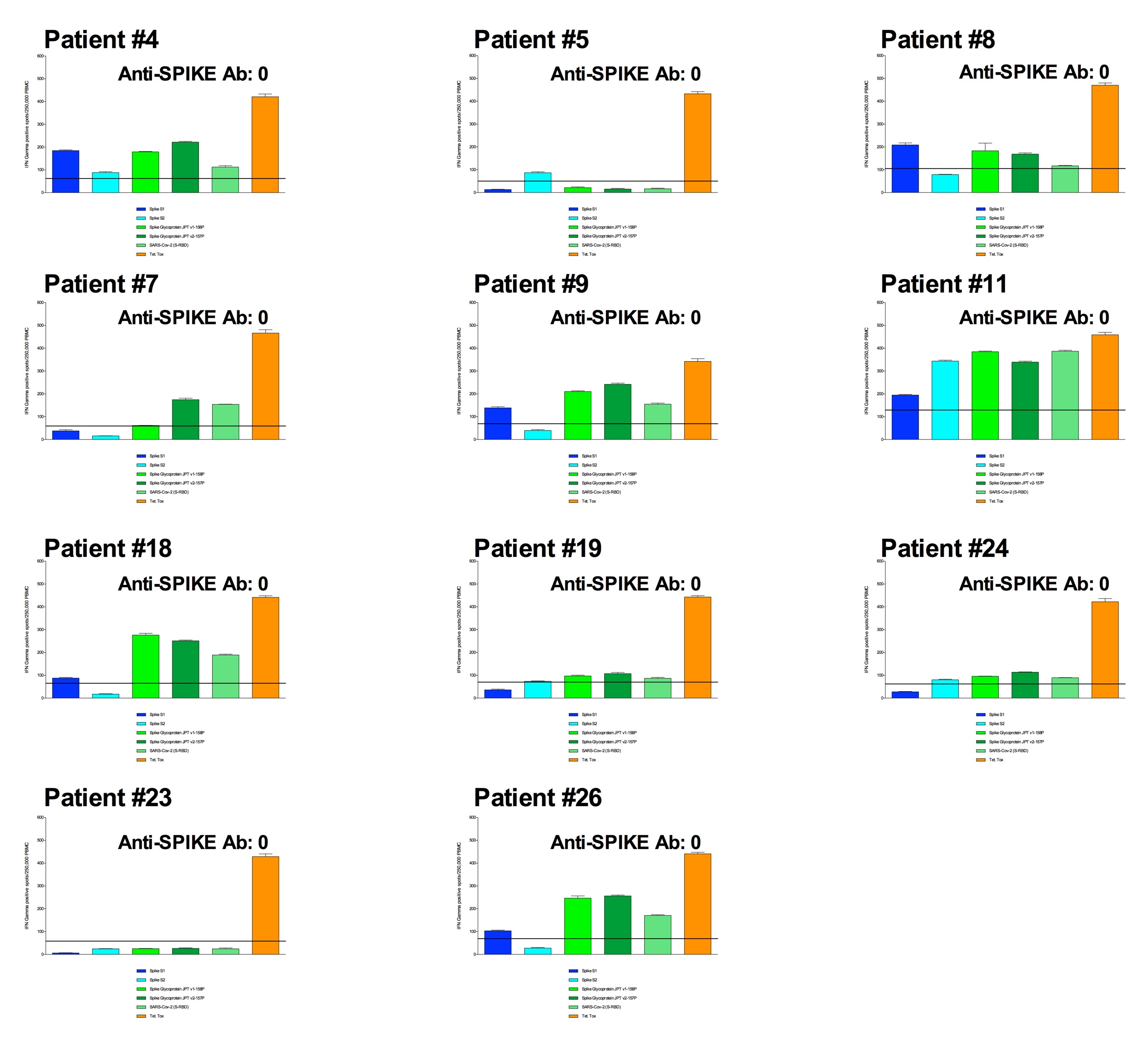

Supplement: Supplementary file 2 [file Image_1.tif]

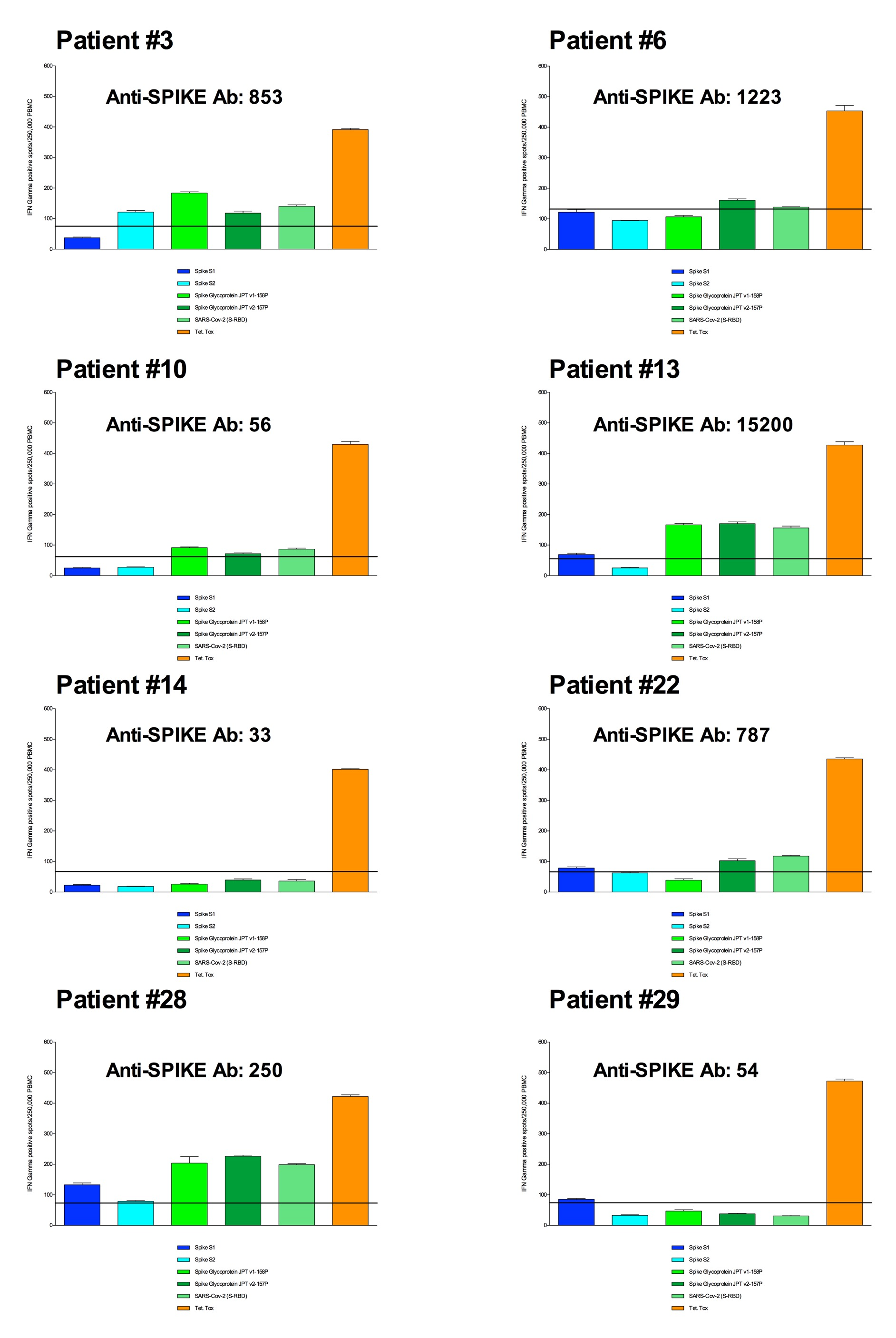

Supplement: Supplementary file 3 [file Image_2.tif]

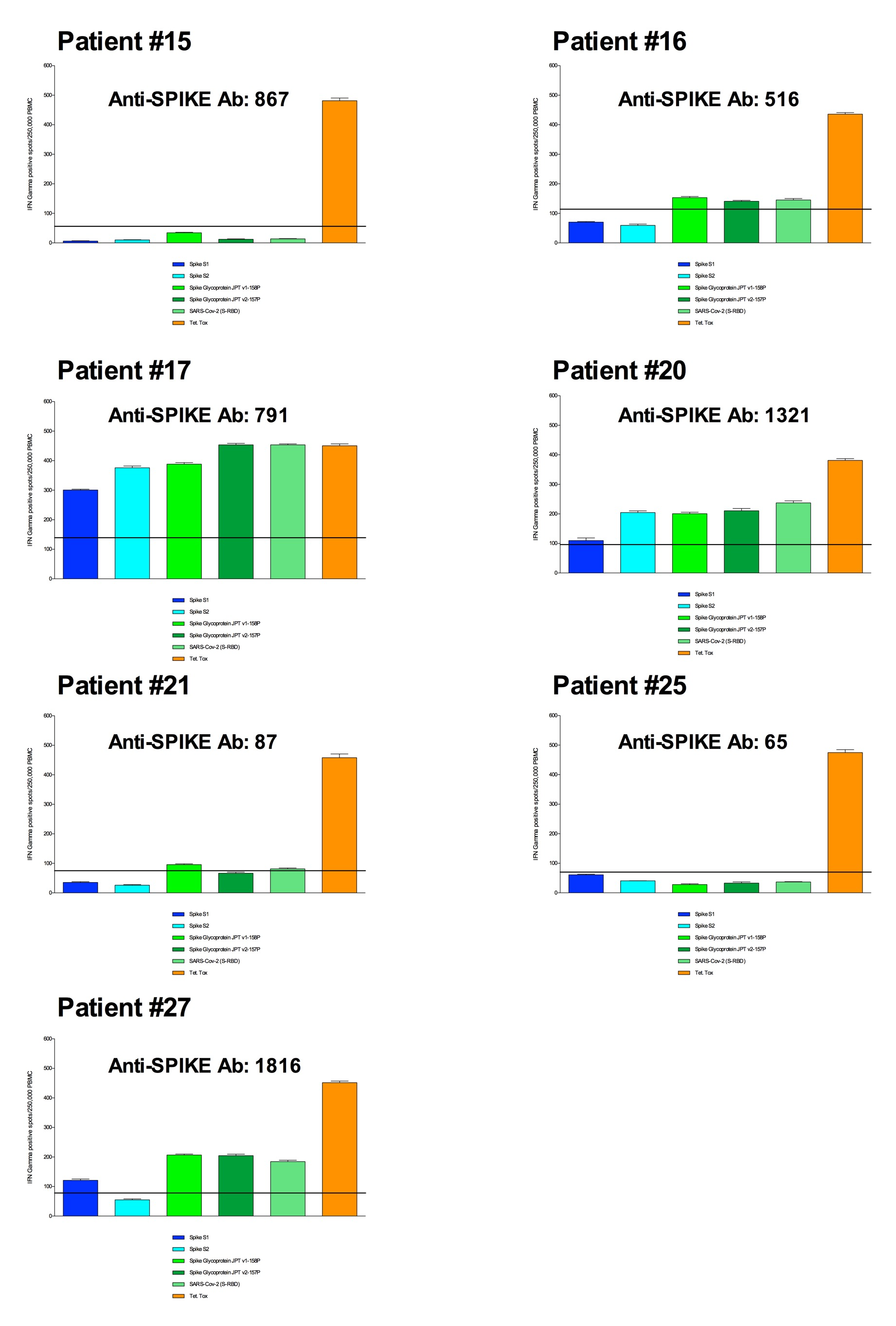

Supplement: Supplementary file 4 [file Image_3.tif]

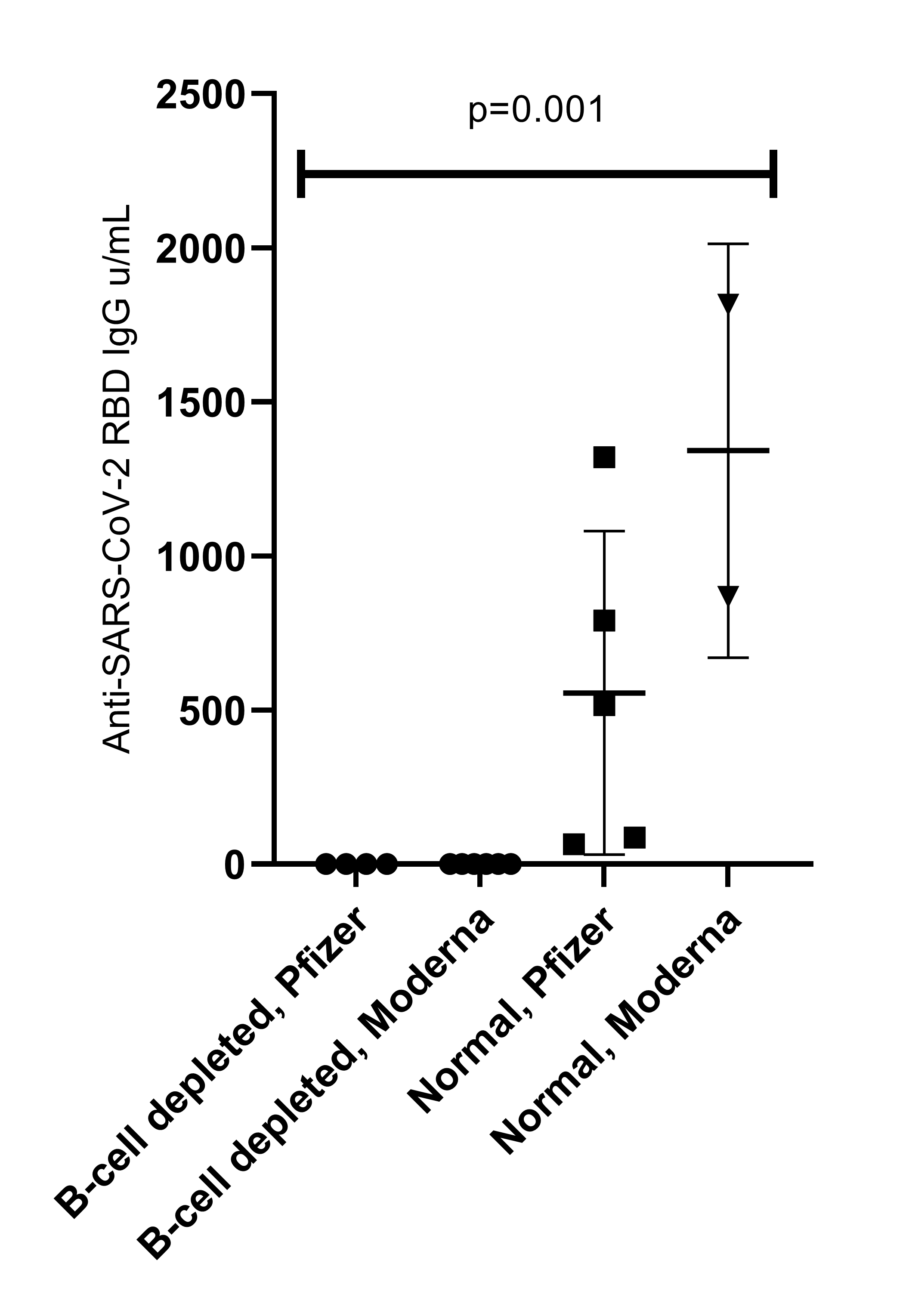

Supplement: Supplementary Figure 4 — Anti-SARS-CoV-2 RBD IgG levels by vaccine type. [file Image_4.tif]

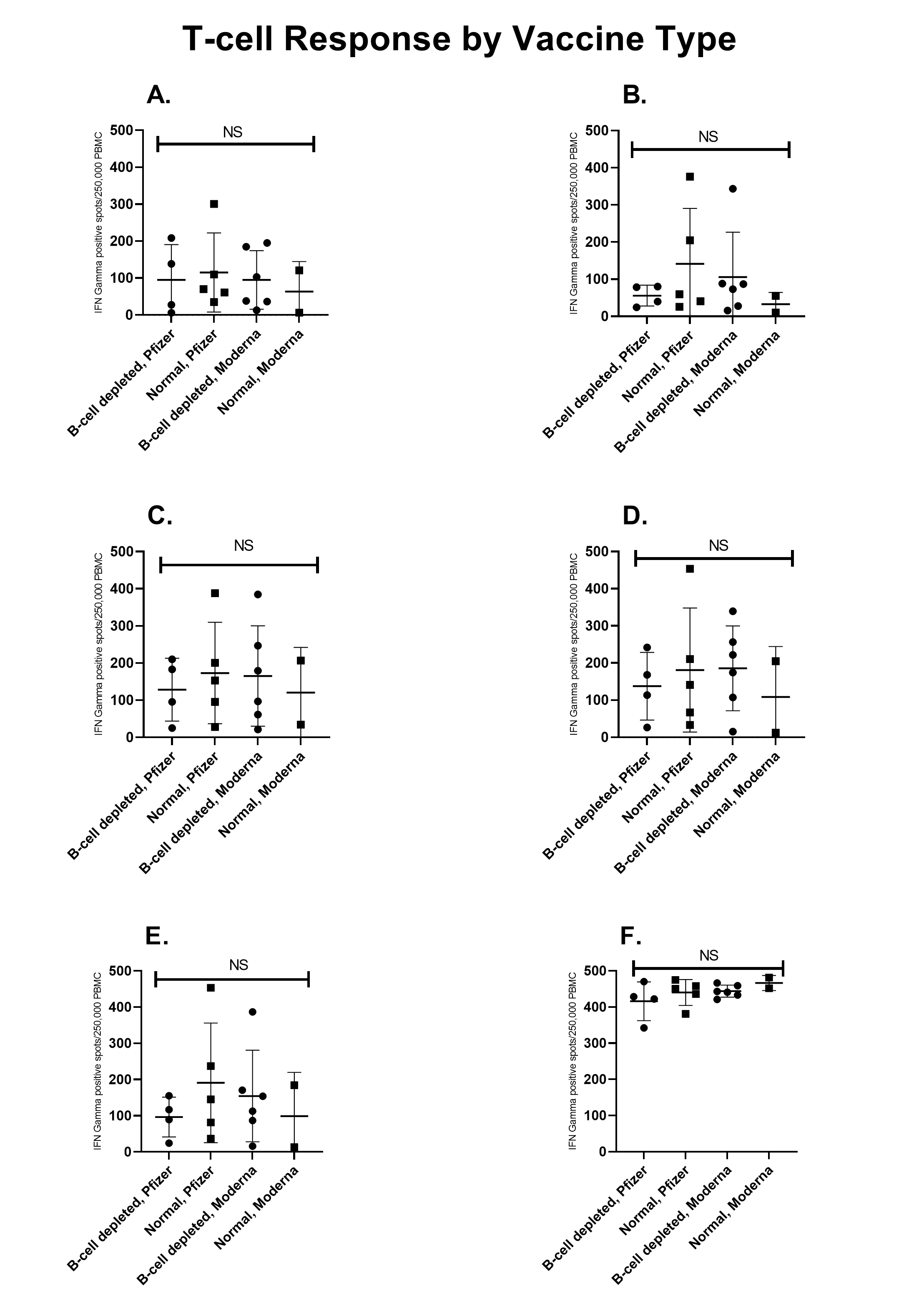

Supplement: Supplementary Figure 5 — Anti-SARS-CoV-2 Spike T-cell responses measured by IFNγ ELISPOT positive spots using different antigens in B-cell depleted patients and normal subjects broken down by vaccine type. (A). S1 subunit SARS-COV-2 spike glycoprotein (B). S2 subunit SARS-COV-2 SPIKE, (C). SARS-COV-2 SPIKE peptide pool (JPT v1-158P), (D). SARS-COV-2 spike peptide pool (JPT v2-157P), (E). SARS-COV-2 spike RBD peptide pool and (F) Tetanus Toxoid. [file Image_5.tif]
